# Phase II Trial of Erlotinib during and after Radiotherapy in Children with Newly Diagnosed High-Grade Gliomas

**DOI:** 10.3389/fonc.2014.00067

**Published:** 2014-04-01

**Authors:** Ibrahim Qaddoumi, Mehmet Kocak, Atmaram S. Pai Panandiker, Gregory T. Armstrong, Cynthia Wetmore, John R. Crawford, Tong Lin, James M. Boyett, Larry E. Kun, Fredrick A. Boop, Thomas E. Merchant, David W. Ellison, Amar Gajjar, Alberto Broniscer

**Affiliations:** ^1^Department of Oncology, St. Jude Children’s Research Hospital, Memphis, TN, USA; ^2^Department of Pediatrics, University of Tennessee Health Sciences Center, Memphis, TN, USA; ^3^Department of Biostatistics, St. Jude Children’s Research Hospital, Memphis, TN, USA; ^4^Department of Radiological Sciences, St. Jude Children’s Research Hospital, Memphis, TN, USA; ^5^Department of Epidemiology and Cancer Control, St. Jude Children’s Research Hospital, Memphis, TN, USA; ^6^Department of Neurosciences, University of California San Diego, San Diego, CA, USA; ^7^Semmes-Murphey Neurologic and Spine Institute, Memphis, TN, USA; ^8^Department of Pathology, St. Jude Children’s Research Hospital, Memphis, TN, USA

**Keywords:** erlotinib, epidermal growth factor receptor, high-grade glioma, pediatric, phase II, radiotherapy

## Abstract

**Background:** Epidermal growth factor receptor is overexpressed in most pediatric high-grade gliomas (HGG). Since erlotinib had shown activity in adults with HGG, we conducted a phase II trial of erlotinib and local radiotherapy (RT) in children with newly diagnosed HGG.

**Methods:** Following maximum surgical resection, patients between 3 and 21 years with non-metastatic HGG received local RT at 59.4 Gy (54 Gy for spinal tumors and those with ≥70% brain involvement). Erlotinib started on day 1 of RT (120 mg/m^2^ per day) and continued for 2 years unless there was tumor progression or intolerable toxicities. The 2-year progression-free survival (PFS) was estimated for patients with intracranial anaplastic astrocytoma (AA) and glioblastoma (GBM).

**Results:** Median age at diagnosis for 41 patients with intracranial tumors (21 with GBM and 20 with AA) was 10.9 years (range, 3.3–19 years). The 2-year PFS for patients with AA and GBM was 15 ± 7 and 19 ± 8%, respectively. Only five patients remained alive without tumor progression. Twenty-six patients had at least one grade 3 or 4 toxicity irrespective of association with erlotinib; only four required dose modifications. The main toxicities were gastrointestinal (*n* = 11), dermatologic (*n* = 5), and metabolic (*n* = 4). One patient with gliomatosis cerebri who required prolonged corticosteroids died of septic shock associated with pancreatitis.

**Conclusion:** Although therapy with erlotinib was mostly well-tolerated, it did not change the poor outcome of our patients. Our results showed that erlotinib is not a promising medication in the treatment of children with intracranial AA and GBM.

## Introduction

High-grade gliomas (HGGs) arising outside the brainstem account for 8–12% of all pediatric central nervous system tumors (1). The 5-year survival for children with glioblastoma (GBM) and anaplastic astrocytoma (AA), the most common types of HGGs, remains <15 and <30%, respectively, despite the use of multi-modality therapies consisting of maximum surgical resection, radiotherapy (RT), and different regimens of chemotherapy (2–4).

The epidermal growth factor receptor (EGFR) belongs to a family of cell-surface proteins that also includes human epidermal growth factor receptors (HER) 2–4. EGFR is activated by binding to its ligands, leading to autophosphorylation of its intracellular tyrosine kinase domain (5). This autophosphorylation activates a downstream cascade of proteins involved in various cell-survival functions. Activation of EGFR is important for tumor survival and growth and is associated with poor clinical outcome in different malignancies (5).

Several EGFR inhibitors have been used for treating different types of cancers, including GBM (5–7). Erlotinib (Tarceva™, OSI Pharmaceuticals, Melville, NY, USA; Roche, Basel, Switzerland; and Genentech, South San Francisco, CA, USA) is an orally active, potent, and selective inhibitor of EGFR and HER2 (8). Early studies showed that *EGFR* amplification is rare in pediatric HGG (9, 10). However, EGFR is overexpressed by immunohistochemistry in 80% of these tumors (11). Preliminary studies showed promising activity of erlotinib against GBM in adult patients (12, 13). In a phase I study of adults with malignant gliomas receiving erlotinib alone or with temozolomide, eight [GBM (*n* = 5), AA (*n* = 1), gemistocytic astrocytoma (*n* = 1), and oligodendroglioma (*n* = 1)] of 57 evaluable patients had a partial response, six of whom received only erlotinib (12). Of these eight patients, four had a progression-free survival (PFS) of more than 6 months, including three patients with GBM. Also, two patients [GBM (*n* = 1) and AA (*n* = 1)] with no tumor response to therapy had a PFS of ≥6 months, including one patient who received only erlotinib.

The preliminary results of clinical trials in adults and the high incidence of EGFR overexpression in pediatric HGGs provided the rationale for conducting a pediatric study using erlotinib to treat children with HGG. Our institution completed a phase I clinical trial, including pharmacokinetic studies, of erlotinib administered during and after RT in children with HGG (14). There were 23 children (12 GBM, 8 AA, and 3 other HGG) treated on our phase I study. The maximum tolerated dose (MTD) of erlotinib was 120 mg/m^2^ per day. Dose limiting toxicities (DLT) in our phase I study were grade 3 diarrhea despite supportive care (*n* = 1 at MTD level), grade 3 increase in serum lipase concomitant with abdominal pain, and grade 3 intolerable skin rash associated with pruritus. The latter two DLT occurred at dose level of 160 mg/m^2^ per day. Use of erlotinib for at least 6 months (*n* = 18) and 12 months (*n* = 12) was well-tolerated with chronic toxicities such as grade 1–3 indirect hyperbilirubinemia, grade 1–3 hypokalemia and hypophosphatemia, and grade 1–3 lymphopenia. Also, one of our patients on the phase I study developed a second neoplasm (rhabdomyosarcoma) outside the radiation field within 6 months of therapy.

We present the results of the phase II study combining RT and erlotinib in children with newly diagnosed, intracranial GBM and AA. Although the outcomes of our patients were not better than those reported in previous clinical trials, our results are relevant since EGFR-targeting agents alone or in combination, particularly erlotinib, continue to be tested in several recent clinical trials on pediatric HGG.

## Patients and Methods

Patients aged 3–21 years with non-metastatic, newly diagnosed HGG or unfavorable low-grade glioma were eligible for this phase II trial (NCT00124657). Unfavorable low-grade gliomas were defined as World Health Organization grade II gliomas with the radiologic diagnosis of gliomatosis cerebri or bithalamic involvement. However, only data from patients with intracranial GBM or AA were included in the analysis of PFS and overall survival (OS). Except for one case, all tumors were centrally reviewed at St. Jude Children’s Research Hospital (St. Jude). Five patients (four with GBM and one with AA) were previously treated at the MTD during the phase I study (14); these patients met the eligibility criteria for the phase II study and were included in this analysis.

Additional eligibility criteria included an interval of ≤42 days between surgery and start of therapy; a performance score ≥40 and adequate organ functions (absolute neutrophil count ≥1000/μL, platelet count ≥100,000/μL, hemoglobin concentration ≥8 g/dL, serum creatinine concentration less than twice the institutional normal values for age, total bilirubin <1.5 times the upper limit of normal, alanine aminotransferase <5 times the institutional upper limit of normal, and albumin ≥2g/dL). Exclusion criteria included diagnosis of diffuse intrinsic pontine glioma, diagnosis of radiation induced HGG, use of enzyme-inducing anticonvulsants, use of other concomitant anticancer treatments, presence of other significant medical problems, and pregnancy or lactation.

The institutional review boards (IRBs) at St. Jude and Rady Children’s Hospital approved the study before initial patient enrollment, and a written informed consent (or assent when appropriate) was obtained from patients or their legal guardians.

### Study design and treatment plan

Patients from St. Jude and Rady Children’s Hospital were enrolled on the study. Following maximum surgical resection, eligible patients received 59.4 Gy local RT administered at a dose of 1.8 Gy per fraction, 5 days per week for 6.5 weeks. The treatment volume encompassed the entire tumor defined on preoperative imaging by the combination of T1, T2-weighted, and FLAIR sequences, a 2-cm margin to account for microscopic disease, and a 0.3- to 0.5-cm margin to account for uncertainty in immobilization and patients’ positioning. The prescribed RT dose was limited to 54 Gy when the required target volume included ≥70% of the whole brain and in patients with spinal tumors.

Since the degree of surgical resection is a major prognostic factor for children with HGG (2), achieving a gross or near-total resection was attempted whenever possible even if more than one surgical procedure was required. Gross total resection was defined as no visible tumor left based on imaging and operative report, and near-total resection was identified as removal of >90% but <100% of the tumor. Subtotal and partial resections were defined as resection of 50–89 and 10–49% of the tumor, respectively. Biopsies were defined as <10% of tumor resection.

Erlotinib was initiated on the first day of RT at the phase II recommended dose of 120 mg/m^2^ per day (maximum dose 200 mg per day) (14). Patients received erlotinib for 26 courses (2 years) without interruption unless there was tumor progression or intolerable toxicities. Each course of erlotinib lasted 28 days.

Table 1 provides the recommendations for dose modifications in case of significant toxicities. All adverse events were graded according to the National Cancer Institute Common Terminology Criteria for Adverse Events (CTCAE) version 3.0. Clinical and radiologic evaluations were similar to those used in the phase I trial (14).

**Table 1:** **Recommended dose modifications in the phase II study**.

Toxicity	Action at first occurrence	Action at recurrence	Grounds for drug discontinuation
Non-hematologic grade 2 (except skin rash and diarrhea)^a^	Hold erlotinib until resolution to ≤grade 1; restart at 90 mg/m^2^ per day	If grade 2, hold erlotinib until resolution to ≤grade 1; restart at 70 mg/m^2^ per day	If recurrent grade 2 toxicity after two dose reductions
Diarrhea and/or skin rash grade 2	Supportive care only; hold erlotinib and restart at 90 mg/m^2^ per day if duration >7 days and toxicity intolerable	Supportive care only; hold erlotinib and restart at 70 mg/m^2^ per day if duration >7 days and toxicity intolerable	If recurrent grade 2 intolerable toxicity lasting >7 days after 2 dose reductions
Non-hematologic grade 3^b^	Hold erlotinib until resolution to ≤grade 2; restart erlotinib at 90 mg/m^2^ per day if resolution ≤7 days. Discontinue erlotinib if grade 3 toxicity persists for >7 days	Discontinue erlotinib if recurrent grade 3 toxicity occurs after 1 dose reduction	If grade 3 toxicity lasts >7 days or if grade 3 toxicity recurs after one dose reduction
Transaminase grade 3 (lasting >7 days)	Hold erlotinib until resolution to ≤grade 1; resume at 90 mg/m^2^ per day	Discontinue erlotinib if recurrent grade 3 toxicity lasts >7 days	If recurrent grade 3 toxicity lasts >7 days
Non-hematologic grade 4^c^	Discontinue erlotinib		
Interstitial lung disease	If suspected, hold erlotinib until disease is ruled out or improved		

*Since hematologic toxicities were not expected with erlotinib, the protocol mandated that the PI to be contacted for grade 3 and 4 hematologic toxicities, prior any holding of modifying the erlotinib dose*.

*^a^Not attributable to another cause and severe enough to warrant treatment discontinuation*.

*^b^Excluding weight gain or loss, diarrhea for ≤48 h not given optimal supportive therapy, tolerable skin rash, fever or non-neutropenic infection, nausea, and vomiting for ≤48 h not given optimal supportive therapy, seizures, elevation of transaminases that resolves to ≤grade 1 within 7 days of discontinuing the drug, or electrolyte abnormalities that resolve to ≤grade 2 within 7 days with or without clinical intervention*.

*^c^Excluding grade 4 electrolyte abnormalities that resolve to ≤grade 2 within 7 days with or without clinical intervention*.

Progression-free survival was defined as the interval between the start of treatment and initial failure, including clinical or radiologic progression, or death from any cause. OS was defined as the interval between the start of treatment and death. Progressive disease (PD) was defined as worsening neurologic status not explained by causes unrelated to tumor progression, or a greater than 25% increase in the product of the maximum perpendicular diameters of any lesions, or increasing doses of corticosteroids required to maintain stable neurologic status.

### Statistical considerations

The primary objective of the current trial was to estimate the PFS for patients with intracranial AA and GBM by the Kaplan–Meier method. The “intent-to-treat” principle was followed, and all eligible patients who received any treatment were included in the analyses. The sequential probability ratio test (SPRT) was used to monitor PFS independently for patients with AA and GBM in order to halt accrual within each stratum as soon as statistical confidence developed that the treatment regimen was not meeting efficacy expectations (15). The SPRT monitoring design for each stratum was based on type 1 and type 2 error rates of 0.10, and monitoring did not begin until five failures had been observed. The fully sequential monitoring rule for patients with AA was based on comparing true and unknown 2-year PFS rates of 25 versus 45%, respectively. The monitoring criteria for patients with GBM were based on comparing true and unknown 2-year PFS rates of 10 versus 25%, respectively. The maximum expected accrual was 29 patients with AA and 30 with GBM. Futility analyses closed accrual for patients with AA and GBM after the 14th and 16th failures, respectively. At that point, 20 patients with AA and 21 with GBM had been accrued to this study. The Fisher’s exact test was used to examine the correlation between dexamethasone use and lymphopenia.

## Results

### Patients’ characteristics

Thirty-nine patients were enrolled on the phase II study between August 2007 and November 2010. Five additional patients treated at the MTD during the phase I trial were incorporated into the current analysis (14). Overall, 20 patients had AA, 21 had GBM, one had anaplastic oligoastrocytoma, one had unfavorable fibrillary astrocytoma, and one had spinal GBM. All patients underwent complete imaging evaluation of the neuraxis before the start of therapy, which showed no evidence of metastatic disease. All 44 patients were included for evaluation of toxicity profiles. Forty-three patients were treated at St. Jude and one at Rady Children’s Hospital.

Table 2 provides the characteristics of 41 patients with intracranial AA and GBM who were eligible for PFS estimation. Thirteen (32%) of these 41 patients had gliomatosis cerebri (*n* = 7; five with AA and two with GBM) or bithalamic tumors (*n* = 6; five with AA and one with GBM).

**Table 2:** **Patients’ characteristics**.

Characteristic	All patients (*n* = 41)	AA (*n* = 20)	GBM (*n* = 21)
**AGE AT DIAGNOSIS (YEARS)**			
Median	10.9	11.8	8.9
Range	3.3–19	3.6–19	3.3–16.6
**LENGTH OF FOLLOW-UP (MONTHS)^a^**			
Median	13.5	16.0	11.7
Range	1.8–66.5	3.9–66.5	1.8–54.8
**SEX**			
Female	24	11	13
Male	17	9	8
**RACE**			
White	30	15	15
Black	7	4	3
Other	4	1	3
**ETHNICITY**			
Non-Hispanic	37	18	19
Hispanic	4^b^	2	2
**LOCATION**			
Cerebrum	22	10	12
Cerebellum	5	1	4
Thalamus	13	9	4
Pineal	1	0	1
**EXTENT OF RESECTION**			
Gross/near-total	10	4	6
Subtotal	12	3	9
Partial/biopsy	19	13	6

*AA, anaplastic astrocytoma; GBM, glioblastoma multiforme*.

*^a^Defined as last contact*.

*^b^Included one of each of the following: Cuban, Mexican, Central/South American, and Spanish not other was specified*.

### Outcomes

**Figure 1:**
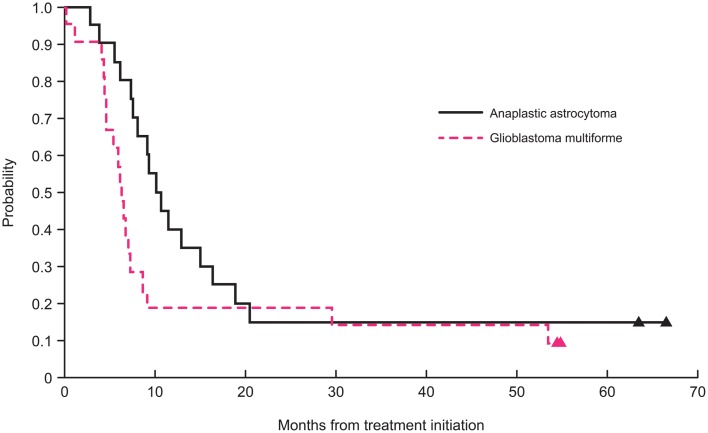
**Two-year progression-free survival curves for patients with glioblastoma and anaplastic astrocytoma**.

After excluding the three non-intracranial GBM or AA cases (one anaplastic oligoastrocytoma, one unfavorable fibrillary astrocytoma, and one spinal GBM), the 2-year PFS was 15 ± 7 and 19 ± 8% for patients with intracranial AA and GBM, respectively (Figure 1). The 2-year OS for patients with AA and GBM was 20 ± 8 and 19 ± 8%, respectively. The 5-year PFS was 15 ± 8 and 9.5 ± 9% for patients with AA and GBM, respectively. The 5-year OS for patients with AA and GBM was 15 ± 10 and 14 ± 13%, respectively. The median PFS for patients with AA and for GBM was 10.7 and 6.3 months, respectively. The median OS for patients with AA and GBM was 15.4 and 11.7 months, respectively.

Two patients died while on study, one each from disease progression and toxicity. The median follow-up for these 41 patients was 13.5 months (range, 1.8–66.5 months).

Thirty-five of 41 patients died (17 AA, 18 GBM), all except one due to disease progression. Five patients (three with AA, two with GBM) remain alive without experiencing disease progression. The median duration of follow-up for these five patients was 55 months (range, 43–66.5 months), of whom three underwent a gross total resection (two with AA and one with GBM), one a subtotal resection (AA), and one a biopsy only (GBM). Two of these five patients discontinued erlotinib due to non-compliance after less than 1 and 12 courses of therapy, respectively. One patient with intracranial GBM who remains alive experienced disease progression 54 months after diagnosis.

### Progressive disease characteristics

Of 35 patients who developed PD, 25 had local progression only, five experienced metastatic failures (three in the spine and two in distant locations within the brain), and five had both local and metastatic PD. Metastatic disease in patients with mixed PD was ventricular (*n* = 3), spinal (*n* = 1), and cerebellar (*n* = 1).

### Toxicities

Twenty-six (59%) of 44 patients developed at least one grade 3 or 4 toxicity irrespective of attribution to erlotinib. Patients received a median of nine courses of chemotherapy (range 1–33). One of the five patients enrolled on the phase I study received 33 courses of chemotherapy since treatment duration was extended for 3 years in the phase I trial (14). The dose of erlotinib was reduced six times in four patients (dose reduction required twice in two patients) due to grade 3 or 4 non-hematologic toxicities (gastrointestinal and dermatologic in three instances each). Treatment with erlotinib was discontinued because of PD (*n* = 32), non-compliance (*n* = 4), or toxicity (*n* = 1). The four non-compliant patients received less than one course (*n* = 1), six courses (*n* = 2), and 12 courses (*n* = 1) of erlotinib. Seven patients tolerated erlotinib well and completed the planned therapy. Table 3 summarizes the toxicities in patients.

**Table 3:** **Toxicity profiles of the study cohort irrespective of attribution to erlotinib**.

Category	Toxicity description	Toxicity grade		Distinct patients for each category
		Grade 3	Grade 4	
Blood	Hemoglobin	2	1	18
	Lymphopenia^a^	7	9	
	Neutrophils	2	1	
	Platelets	–	1	
Dermatologic	Pruritus	3	–	5
	Rash/desquamation	1	–	
	Rash/acne	2	–	
Gastrointestinal	Anorexia	3	–	11
	Diarrhea	5	–	
	Dysphagia	1	–	
	Mucositis	1	–	
	Nausea	2	–	
	Vomiting	4	–	
Metabolic	ALT/AST	2	–	4
	Hypokalemia	2	–	
	Bilirubin	1	–	
Pain^b^	Headache	1	1	2
Constitutional	Fatigue	1	–	3
	Weight loss	2	–	
Total distinct number of patients				26

*^a^Eight of the 16 patients with lymphopenia received dexamethasone within 4 weeks of the recorded toxicity*.

*^b^There was a documented progressive disease within 3 days of the recorded headache*.

The only treatment**-**related mortality occurred in a 12.9-year-old African-American female with extensive gliomatosis cerebri (AA) who was on chronic use of corticosteroids for 44 weeks (30 weeks before study enrollment). Erlotinib was held in this patient after 73 days of treatment because of diarrhea. Three days later, laboratory tests and imaging confirmed pancreatitis. Peak serum levels of lipase and amylase were 892 units/L (grade 4 toxicity) and 375 units/L (grade 3 toxicity), respectively. Her lowest total and ionized calcium concentrations were 6.9 and 1.1 mmol/L, respectively. Her peak total and direct bilirubin concentrations were 8.9 and 6.2 mg/dL, respectively. She also developed a wound infection at the craniotomy site. She subsequently developed septic shock and died 99 days after the start of therapy. An autopsy was not performed. It was impossible to determine the exact cause of the septic shock in this patient, but we believe that the pancreatitis was the main contributing factor.

## Discussion

Unlike for adults with HGG (16), no chemotherapy regimen is considered part of standard care for children with newly diagnosed HGG in North America. When our phase II trial was designed, temozolomide during and/or after local RT did not improve the outcome of children with AA and GBM (3, 4). The failure of temozolomide to improve the outcome of children with HGG, the common overexpression of EGFR in pediatric HGG, and the preliminary promising activity of erlotinib against recurrent GBM in adults prompted us to conduct this trial (3, 4, 11–13).

We showed that the use of erlotinib during and after RT did not improve the poor outcome of children with newly diagnosed intracranial AA and GBM. Although five patients were long-term survivors and free of tumor progression, including one who received less than 1 course of erlotinib, the majority had at least one characteristic at diagnosis associated with a better outcome (e.g., the diagnosis of AA and the presence of no residual disease at the start of therapy). On the other hand, a large majority of our 41 patients had poor prognostic factors before the start of therapy, including less than a gross total/near-total resection (76%), presence of gliomatosis cerebri (17%), or tumors arising from one (17%) or both thalami (15%) (17, 18). Interestingly, the majority of the gliomatosis cerebri cases (5 of 7) and bithalamic tumors (5 of 6) were patients with AA tumors, and this could explain the similar outcome to GBM patients in our cohort.

Three phase I trials reported the safety, pharmacokinetics, and MTD of erlotinib alone or in combination in children with newly diagnosed or progressive/recurrent brain and solid tumors (14, 19, 20). In one study, two patients, one with a malignant glioma and one with an anaplastic oligoastrocytoma, experienced a 44 and 47% tumor regression after therapy with erlotinib alone, respectively (20).

Similar to our phase I trial (14), we incorporated detailed, optional plasma pharmacokinetic analysis in the current study. Unfortunately, adherence to these studies was very low in the phase II trial, which precluded any meaningful analysis.

Erlotinib was in general well-tolerated by our patients. Only four (9%) patients required dose modification due to toxicities, specifically significant diarrhea and skin rash. Other grade 3 and 4 toxicities were uncommon and were mainly pruritus, anorexia with weight loss, nausea, and vomiting, indirect hyperbilirubinemia, increase in liver function tests, and hypokalemia. These toxicities were very similar to those described in both phase II adult and phase I pediatric trials using erlotinib as a single agent (14, 19–22). To explain the high rate of lymphopenia in our study, we examined the correlation between the use of dexamethasone and onset of lymphopenia. One patient who was treated outside of St. Jude was not included in the analysis due to lack of dexamethasone information. There were 16 patients who had at least one episode of grade 3/4 lymphopenia in this study. Eight of them (8/16, 50%) received dexamethasone as concomitant treatment. Among the other 27 patients, 9 of them (9/27, 33%) received dexamethasone. The Fisher’s exact test has *p*-value = 0.34. The concomitant use of dexamethasone might promote the development of lymphopenia in some of the patients, but in general, we do not have sufficient evidence to say it is associated with lymphopenia in this cohort.

Although one patient died of septic shock, both the chronic use of steroids and the pancreatitis that preceded this event likely contributed to her death. Interestingly, one patient who received erlotinib at 160 mg/m^2^ developed a grade 3 increase in serum lipase without radiologic findings suggestive of pancreatitis in the phase I study (14). There is only one report of fatal pancreatitis in a 46-year-old patient with non-small-cell lung cancer receiving erlotinib (23). However, in that case erlotinib was combined with sunitinib, which is reported to cause grade 3 and 4 increases in serum amylase and lipase in 5 and 16% of adult patients with renal carcinoma, respectively (24).

Several studies in adults with GBM suggested that specific tumor markers are associated with a better response to small-molecule inhibitors of EGFR, including erlotinib (13, 25). Although a recent study showed a slightly higher occurrence of *EGFR* amplification and/or mutations in children with HGG (26), larger recent genome-wide molecular analyses demonstrated that these changes occur in only a few cases (27, 28). Although we performed extensive molecular analysis in the phase I study (14), we did not perform these studies in the current trial because of the poor outcome of patients. Likewise, a more recent randomized clinical trial in adults with progressive GBM showed that erlotinib less effective than temozolomide and lomustine (21).

In summary, similar to previous studies using other chemotherapy regimens, we showed that the use of erlotinib during and after local RT did not change the poor outcome of children with intracranial AA and GBM. The advances made in elucidating the molecular mechanisms regulating the genesis of pediatric HGG (27–29) will hopefully lead to the use of targeted therapies that are more suitable to the biologic characteristics of these cancers.

## Conflict of Interest Statement

All study medications were provided by OSI Pharmaceuticals Inc. and Genentech Inc. Dr. Alberto Broniscer received partial financial support from OSI Pharmaceuticals Inc. and Genentech Inc. to conduct this study. The other co-authors reports no conflicts of interest.
